# Hsa-miR-99a deficiency contributes to MSI-H colorectal cancer progression by activating the mTOR pathway and inducing Th1/Th2 imbalance

**DOI:** 10.3389/fimmu.2026.1796084

**Published:** 2026-03-17

**Authors:** Xuejing Yang, Tingting Zhang, Hu Sun, Huijing Feng, Dong Song

**Affiliations:** 1 Department of Thoracic Oncology, Cancer Center, Shanxi Bethune Hospital, Shanxi Academy of Medical Sciences, Tongji Shanxi Hospital, Third Hospital of Shanxi Medical University, Taiyuan, Shanxi, China; 2 Department of Lymphoma, Cancer Center, Shanxi Bethune Hospital, Shanxi Academy of Medical Sciences, Tongji Shanxi Hospital, Third Hospital of Shanxi Medical University, Taiyuan, Shanxi, China

**Keywords:** colorectal cancer, Hsa-miR-99a, immune infiltration, microsatellite instability, mTOR pathway

## Abstract

**Background:**

Hsa-miR-99a has been linked to the advancement of several malignancies, including colorectal cancer (CRC). This investigation seeks to elucidate its function and regulatory network in CRC.

**Methods:**

Differential expression of hsa-miR-99a was analyzed between microsatellite stable (MSS) and microsatellite instability-high (MSI-H) subgroups. Potential target mRNAs of hsa-miR-99a were retrieved from TargetScan and miRWalk databases. Overlapping mRNAs were subjected to correlation analysis, with candidate genes selected based on |correlation coefficient| > 0.3 and p < 0.05. Enrichment analyses were performed, highlighting key pathways, particularly mTOR signaling. Immune infiltration profiles were compared between MSS and MSI-H groups. Correlation between hsa-miR-99a and mTOR pathway-related genes was validated by RT-qPCR and Western blot. Additionally, multiplex immunohistochemistry (mIHC) with tyramine signal amplification (TSA) was used to characterize the immune microenvironment in MSI-H CRC samples.

**Results:**

Hsa-miR-99a expression was significantly higher in MSS compared to MSI-H groups. Twelve key target genes were identified, including ATP2B2, ATP2B4, SLC8A1, KCNJ5, NTRK3, TNFRSF19, GHR, CXCL12, NTNG1, SDC2, SFRP1, and PRICKLE2. Immune infiltration analysis revealed significant differences in 24 cell types between MSS and MSI-H groups, including Th1, Th2, Th17, and effector memory CD8+ T cells. Ten mTOR pathway-related genes (ATP6V1G2, WNT2, WNT6, WNT9A, WNT9B, FZD1, FZD4, FZD8, IGF1, AKT3) exhibited strong correlation with hsa-miR-99a. Among these, ATP6V1G2 and WNT6 were upregulated in the MSI-H group. mIHC analysis indicated reduced Th1 but increased Th2 and Th17 biomarkers in MSI-H CRC.

**Conclusion:**

This study identified key genes and immune microenvironment alterations regulated by hsa-miR-99a in CRC, offering novel insights and potential therapeutic targets for CRC treatment.

## 1 Introduction

Colorectal cancer (CRC) is the third most frequent tumor and claims the second-highest number of cancer deaths, making it a major worldwide health challenge (1, 2). Its pathogenesis follows the classic “adenoma-carcinoma progression” sequence, involving the accumulation of genetic and epigenetic alterations that result in uncontrolled cellular proliferation and metastasis (3, 4). A critical molecular classification of CRC is based on microsatellite instability (MSI) status, which arises from deficient DNA mismatch repair (5). Tumors characterized by high MSI (MSI-H) exhibit a distinct hypermutated genotype, abundant tumor-infiltrating lymphocytes, and a unique immunogenic profile compared to microsatellite stable (MSS) tumors (6). This molecular difference directly leads to divergent clinical responses: MSI-H CRC demonstrates differential responses to conventional chemotherapy and notable sensitivity to immune checkpoint inhibitors (ICIs), while MSS tumors, which comprise the majority, remain largely refractory to immunotherapy, highlighting the urgent need for novel therapeutic targets (7–10). Consequently, understanding the specific molecular drivers underlying different MSI subtypes is essential for tailoring more precise and effective CRC therapies.

MicroRNAs (miRNAs) are small non-coding RNAs that modulate gene expression after transcription: they latch onto specific mRNAs to either speed up their breakdown or block their translation (11). The hsa-miR-99a exerts a dual regulatory role in CRC, functioning as both a tumor suppressor and an oncogene. Specifically, hsa-miR-99a directly targets the 3’-untranslated region (3’-UTR) of the mTOR gene, thereby inhibiting mTOR protein expression, blocking the mTOR signaling pathway, reducing the phosphorylation of downstream proteins (e.g., p70S6K), and ultimately suppressing the proliferation, migration, and invasion of CRC cells (12). On the other hand, extracellular vesicles (EVs) secreted by CRC cells deliver miR-99a into normal fibroblasts (NFs), where it targets and downregulates NLRP2 expression. This abrogation of NLRP2-mediated inhibition on the NF-κB pathway promotes the transformation of NFs into cancer-associated fibroblasts (CAFs). Activated CAFs secrete CCL7, which induces epithelial-mesenchymal transition (EMT) in CRC cells via the CCR5-mTOR-p70S6K pathway; concurrently, TGF-β1 secreted by CAFs upregulates miR-99a expression in CRC cells, forming an intercellular positive feedback loop that sustains metastatic progression (13). Additionally, miR-99a can target FGFR3 to repress its expression, thereby blocking the FGFR3-Ras-MAPK pathway and inhibiting CRC cell proliferation and differentiation (14). In CRC, hsa-miR-99a is frequently dysregulated and plays a role in regulating tumor cell proliferation, apoptosis, and metastasis by targeting key signaling pathways such as mTOR (15, 16). The mTOR pathway acts as a key control hub for cellular growth, metabolic activity, and survival, with its aberrant activation being a hallmark of CRC progression (17). However, the regulatory network of hsa-miR-99a in CRC, its expression and functional differences across MSI subtypes, and its impact on the tumor immune microenvironment (TIME) remain insufficiently understood.

To systematically elucidate the specific mechanisms of hsa-miR-99a in MSI-associated CRC subtypes, this study conducted a comprehensive investigation integrating bioinformatics analysis with experimental validation. Initially, this study analyzed expression differences of hsa-miR-99a between MSS and MSI-H subtypes and systematically predicted its downstream target genes. Immune infiltration analysis was performed to characterize the immune microenvironment across these subtypes and assess its potential association with hsa-miR-99a-regulated genes. Further experimental validation of key mTOR pathway molecules was carried out using reverse transcription-quantitative polymerase chain reaction (RT-qPCR) and Western blotting. Additionally, TIME of the MSI-H subtype was visualized and quantitatively analyzed using multiplex immunohistochemistry (mIHC) with tyramine signal amplification (TSA). This study aims to provide a comprehensive understanding of hsa-miR-99a’s role and its regulatory network in CRC (with a particular focus on the MSS subtype), offering a scientific foundation for advancing the understanding of its molecular mechanisms and identifying potential therapeutic targets.

## 2 Materials and methods

### 2.1 Data acquisition

The Cancer Genome Atlas (TCGA)-COAD and TCGA-READ transcriptomic profiles along with matched clinical metadata were retrieved from Genomic Data Commons Data Portal (https://portal.gdc.cancer.gov/), which included transcriptome profiles from 51 normal colorectal tissue samples and 618 primary CRC tissue samples. Expression data for hsa-miR-99a (mature miRNA) from these samples were retrieved from the TCGA miRNA-seq datasets. Based on available clinical classification, samples were categorized into MSS (n = 421) and MSI-H (n = 81) groups for comparative analysis. A single-cell RNA sequencing (scRNA-seq) dataset (GSE161277), generated on the GPL20795 platform and containing 4 paired normal and CRC tissue samples, was retrieved from the Gene Expression Omnibus (GEO) database (https://www.ncbi.nlm.nih.gov/geo/).

### 2.2 Differential expression and clinical correlation analysis

Differential hsa-miR-99a expression between the MSS and MSI-H groups was assessed using the Wilcoxon rank-sum test (*P* < 0.05). To assess its prognostic value, CRC samples were dichotomized into high and low hsa-miR-99a expression groups using the optimal cutoff computed with the surv_cutpoint function of the R package “survminer” (v0.4.9). Kaplan-Meier curves were plotted and survival disparities assessed by log-rank test. Associations between hsa-miR-99a expression levels and various clinical parameters (including gender, age, tissue type, and TNM stage) were examined using Chi-square test or Wilcoxon test, as appropriate.

### 2.3 Identification and functional analysis of candidate target genes

Experimentally validated and predicted mRNA targets of hsa-miR-99a (hsa-miR-99a-5p and hsa-miR-99a-3p) were retrieved from the TargetScanHuman (v8.0) and miRWalk (v3.0) databases. The intersection of targets from both databases was used to generate a high-confidence list. Spearman’s correlation between hsa-miR-99a and the intersected mRNAs was calculated across all TCGA-CRC samples. Genes with |r| > 0.3 and *P* < 0.05 were retained as candidate target genes, and their functional landscape was then surveyed with Gene Ontology (GO) and Kyoto Encyclopedia of Genes and Genomes (KEGG) enrichment via “clusterProfiler” (v4.6.2), adopting an adjusted *P* < 0.05. The genes enriched in the KEGG pathways are designated as the key genes for downstream analysis. To construct a potential regulatory network, long non-coding RNAs (lncRNAs) interacting with hsa-miR-99a were predicted using the starBase database (v3.0). A key gene–miRNA–lncRNA regulatory network was visualized using Cytoscape software (v3.10.2).

### 2.4 Immune infiltration analysis

The relative abundance of 24 immune cell types in each CRC sample was quantified using ssGSEA algorithm. Differences in immune cell infiltration scores between the MSS and MSI-H groups were tested with Wilcoxon rank-sum test. Additionally, Spearman correlation analysis was conducted to evaluate the relationships between the infiltration levels of specific immune cells, hsa-miR-99a expression, and the identified key genes.

### 2.5 Single-cell analysis

The scRNA-seq data from the GSE161277 dataset were processed and analyzed using the R package “Seurat” (v5.1.0). Low-quality cells were discarded if they expressed < 200 or > 5,000 genes or with mitochondrial genes exceeded 20% of total counts, and genes detected in fewer than 3 cells. Data normalization, identification of highly variable genes (top 2,000 genes using the ‘vst’ method), scaling, and principal component analysis (PCA) were performed following the standard Seurat workflow. Subsequently, a graph-based clustering algorithm with a resolution of 0.2 was applied to the first 30 principal components to identify cell clusters, and then visualized using UMAP. Cell types were manually annotated by referring to the CellMarker database and standard marker genes from published literature (18). The cell-cell communication network was inferred and analyzed using the “CellChat” package (v1.6.1), with the ligand-receptor database set to CellChatDB.human to accurately predict cell-to-cell signal interactions. For major cell populations, secondary sub-clustering was performed to identify key subtypes, and the expression patterns of key genes across these subtypes were examined. Pseudotime trajectory analysis of relevant cell lineages was conducted using the “Monocle2” package (v2.26.0), and pathway activity scores for key KEGG pathways were calculated using the “AUCell” package (v1.20.2).

### 2.6 Analysis of mTOR pathway interaction

A gene set related to the mTOR signaling pathway (KEGG pathway ID: hsa04150), comprising 157 genes, was downloaded from the KEGG database. Spearman correlation analysis was performed between the expression of each mTOR pathway gene and hsa-miR-99a across the TCGA-CRC cohort. Genes with |r| > 0.3 and p < 0.05 were defined as mTOR-hsa-miR-99a interaction genes.

### 2.7 Clinical sample collection

Peripheral blood samples and matched formalin-fixed, paraffin-embedded (FFPE) tumor tissue samples were collected from patients with CRC diagnosed at Shanxi Bethune Hospital between 2022 and 2025. The Institutional Review Board of The Research Ethics Committee of Shanxi Bethune Hospital granted approval (Ethics Approval Number: SBQKL-2022-057), and every participant provided signed informed consent. MSI status was determined clinically using PCR or IHC.

### 2.8 RT-qPCR

Total RNA, including miRNA, was extracted from peripheral blood samples using TRIzol reagent (Servicebio, G3013) or a dedicated miRcute miRNA Isolation Kit (Tiangen, DP501), following manufacturers’ protocols. For mRNA analysis, cDNA was synthesized using a reverse transcription kit (Abclonal). For miRNA analysis, cDNA was synthesized using the miRcute Plus miRNA First-Strand cDNA Kit (Tiangen, KR211). RT-qPCR was performed using Genious 2X SYBR Green Fast qPCR Mix (Abclonal, RK21203) for mRNAs and the miRcute Plus miRNA qPCR Kit (Tiangen, FP411) for miRNAs on a QuantStudio system. Gene expression was quantified using the 2^-ΔΔCt^ method, with GAPDH and U6 snRNA serving as endogenous controls for mRNA and miRNA, respectively. All primer sequences are listed in **Supplementary Table 1**.

### 2.9 Protein extraction and western blotting

Total protein was extracted from whole blood samples using RIPA lysis buffer (Beyotime, P0013B) supplemented with 1% PMSF (Servicebio, CR2404006). Protein concentrations were measured using a BCA assay. Equal amounts of protein (e.g., 30 μg) were separated by SDS-PAGE and transferred to PVDF membranes. After blocking with 5% non-fat milk, membranes were incubated overnight at 4 °C with specific primary antibodies: anti-AKT3 (1:1000, Abclonal, A22738), anti-ATP6V1G2 (1:1000, Abclonal, A20163), anti-WNT6 (1:1000, Abways, BY2795), anti-mTOR (1:1000, Proteintech, 81670-1-RR), and anti-GAPDH (1:5000, Abclonal, AC001). Following incubation with an HRP-conjugated secondary antibody (Invitrogen, 31460, 1:5000), protein bands were visualized using an ECL chemiluminescence substrate and quantified with ImageJ software.

### 2.10 mIHC with TSA

To characterize the immune microenvironment, multiplex fluorescent IHC was performed on FFPE CRC tissue sections using an Opal TSA multiplex kit (Servicebio). Briefly, 4 μm tissue sections were dewaxed, rehydrated, and subjected to antigen retrieval in citrate buffer (pH 6.0). Endogenous peroxidase was then inactivated. Sequential rounds of staining were carried out for each target biomarker: sections were successively incubated with primary antibody, HRP-conjugated secondary antibody, and Opal fluorophore. After each round, the antibody complex was stripped *via* microwave treatment before the next cycle. Nuclei were counterstained with DAPI. Stained slides were scanned using a multispectral imaging system (Vectra/Polaris, Akoya Biosciences), and images were analyzed using inForm or QuPath software for cell phenotyping and quantification.

### 2.11 Statistical analysis

Bioinformatics analyses were executed in R software (v4.3.0); two-group contrasts were evaluated with Wilcoxon rank-sum test, unless otherwise specified. Data were expressed as the mean ± standard deviation (SD) of three independent experiments with 3 or more independent bio logical replicates. For the comparison of differences between 2 groups, Student’s t-test was used. For comparison of differences between multiple groups, one-way ANOVA and Tukey *post hoc* multiple comparisons were used to determine statistical differences. A *P* value < 0.05 was considered statistically significant for all analyses.

## 3 Results

### 3.1 Differential expression and clinical relevance of hsa-miR-99a in MSS and MSI-H CRC

**Figure 1:**
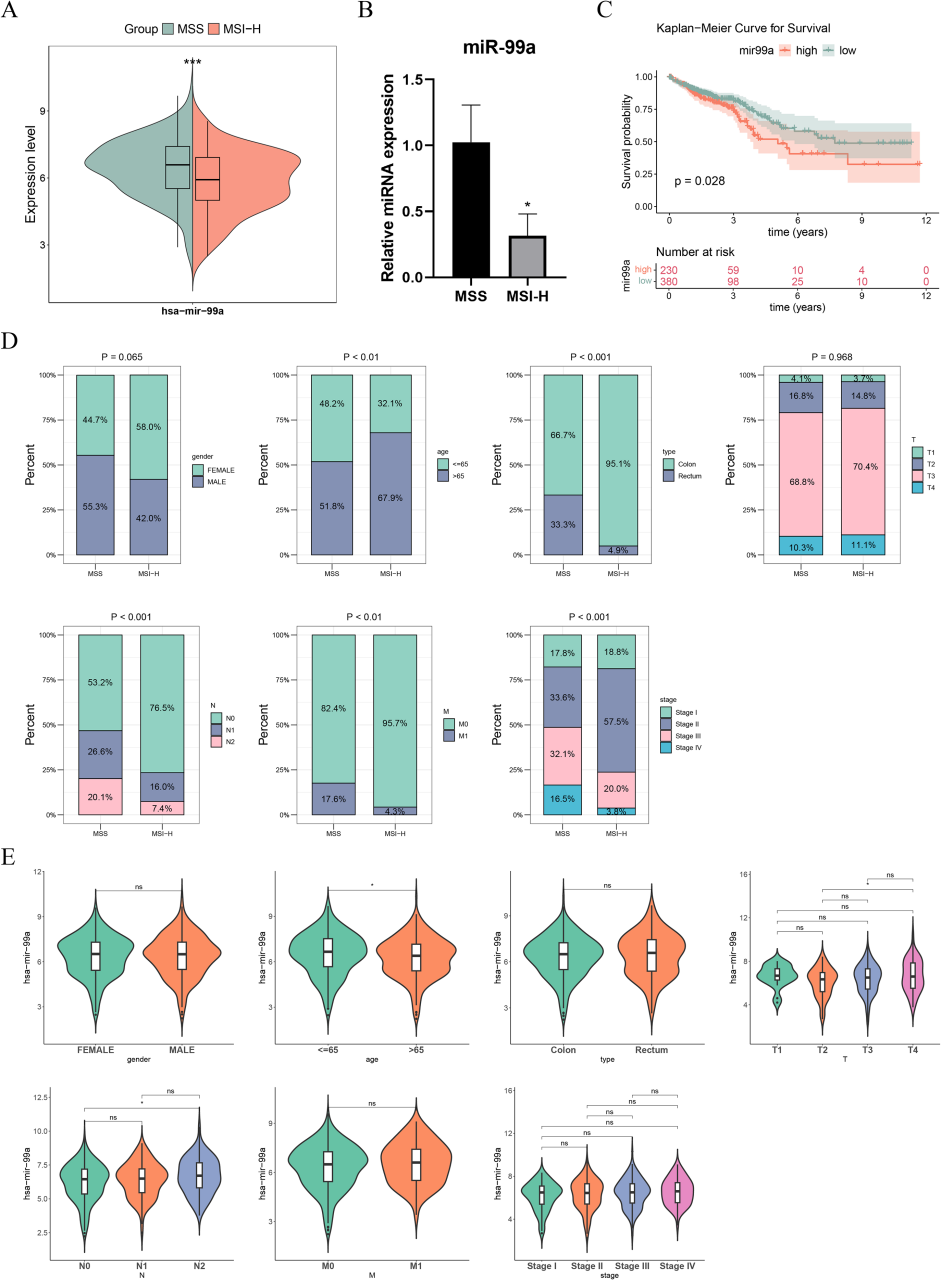
Expression and clinical significance of hsa-mir-99a in MSS and MSI-H groups. **(A)** The differential expression of hsa-mir-99a between microsatellite stable (MSS) and microsatellite instability-high (MSI-H) groups. **(B)** The expression of hsa-mir-99a between MSS and MSI-H CRC blood samples. **(C)** Survival difference between high and low hsa-mir-99a expression groups. **(D)** The differential proportion of clinical features between MSS and MSI-H. **(E)** The differential expression of hsa-mir-99a across differential clinical features. Ns, no significance, *P < 0.05, ***P < 0.001.

Analysis of the TCGA-CRC cohort revealed significantly higher expression of hsa-miR-99a in MSS tumors compared to MSI-H tumors (*P* < 0.05) (**Figure 1A**). This pattern was validated in our cohort of patients with CRC, where elevated hsa-miR-99a levels were observed in the MSS group (**Figure 1B**). Using an expression-optimized cutoff, CRC samples were spilt into high and low hsa-miR-99a expression groups, KM curves revealed markedly poorer overall survival in the high-expression cohort (*P* < 0.05) (**Figure 1C**). Examination of clinical parameters showed significant differences in age, tissue type, M stage, N stage, and overall pathological stage between the MSS and MSI-H groups (**Figure 1D**). Furthermore, hsa-miR-99a expression varied significantly across different age groups and T/N stages (**Figure 1E**).

### 3.2 Identification of 12 key genes regulated by hsa-miR-99a

**Figure 2:**
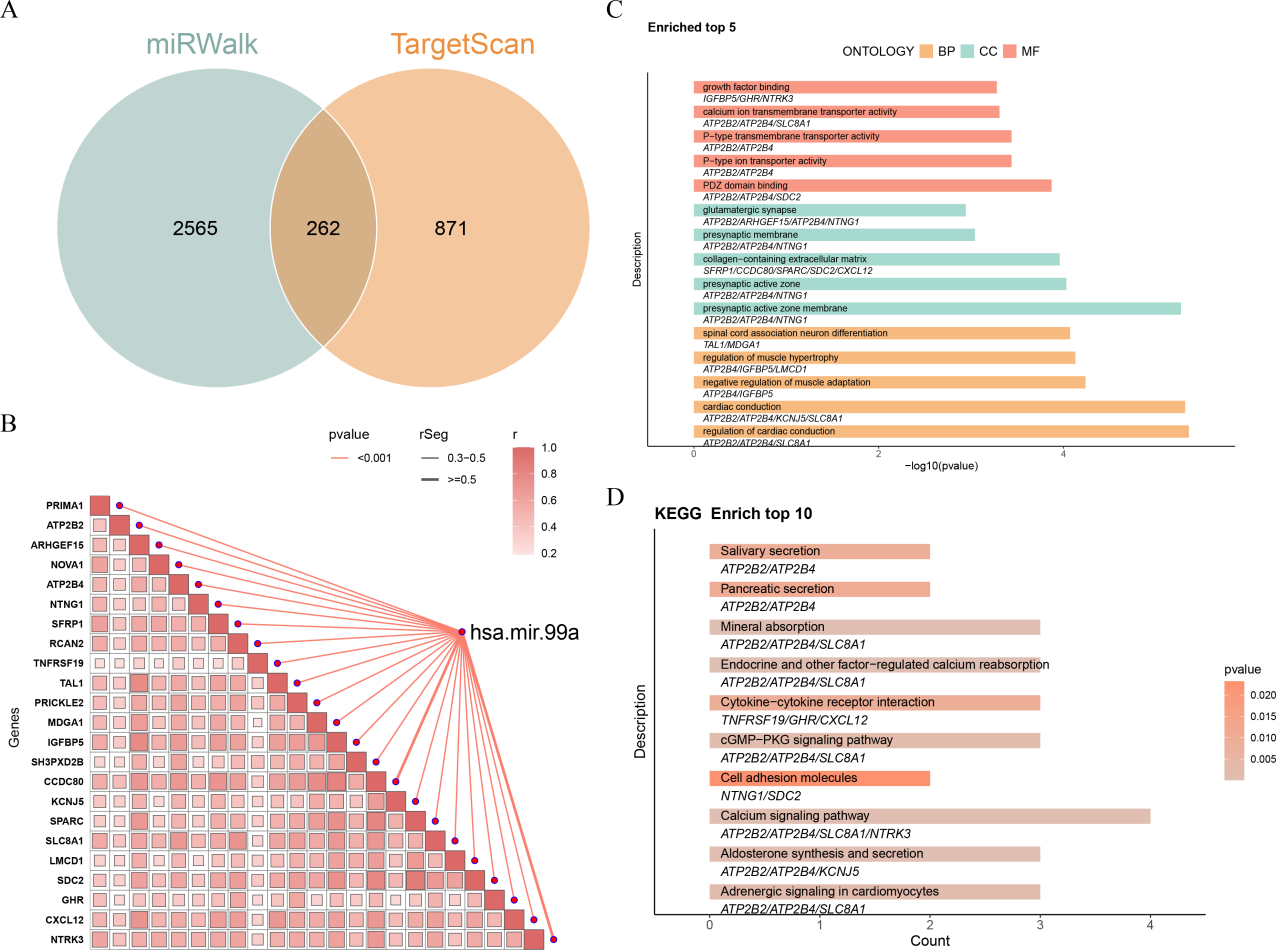
A total of 12 key genes were identified. **(A)** The intersection of mRNAs derived from miRWalk and TargetScan databases. **(B)** The correlation of hsa-mir-99a with mRNAs. **(C, D)** Gene Ontology (GO) terms **(C)** and Kyoto Encyclopedia of Genes and Genomes (KEGG) pathways **(D)** of candidate genes.

To pinpoint the genes functionally regulated by hsa-miR-99a, its predicted mRNA targets were retrieved from TargetScan and miRWalk databases, revealing 262 overlapping genes (**Figure 2A**). Spearman correlation analysis between these genes and hsa-miR-99a expression in the TCGA-CRC dataset identified 23 candidate genes (|r| > 0.3, p < 0.05), including PRIMA1 and ATP2B2 (**Figure 2B**). Functional enrichment analysis of these candidate genes identified 481 significant GO terms and 13 KEGG pathways (**Figures 2C, D**). Enriched GO terms included “regulation of cardiac conduction” and molecular functions such as “PDZ domain binding.” Key enriched KEGG pathways included the “Calcium signaling pathway,” “cGMP-PKG signaling pathway,” and “Adrenergic signaling in cardiomyocytes.” Among the 13 pathways, 12 genes (ATP2B2, ATP2B4, SLC8A1, KCNJ5, NTRK3, TNFRSF19, GHR, CXCL12, NTNG1, SDC2, SFRP1, and PRICKLE2) were selected as the key genes for downstream analysis. The relevant information about genes and pathways can be found in **Supplementary Table 2**. A comprehensive regulatory network integrating these key genes, hsa-miR-99a (5p and 3p strands), their predicted interacting lncRNAs, and associated pathways was constructed, comprising 50 nodes and 70 interaction pairs (**Supplementary Figure 1**). Notable interactions within this network included NTRK3-hsa-miR-99a-5p-LINC01128 and ATP2B2/NTNG1-hsa-miR-99a-3p.

### 3.3 Distinct immune cell infiltration landscapes in MSS and MSI-H CRC

**Figure 3:**
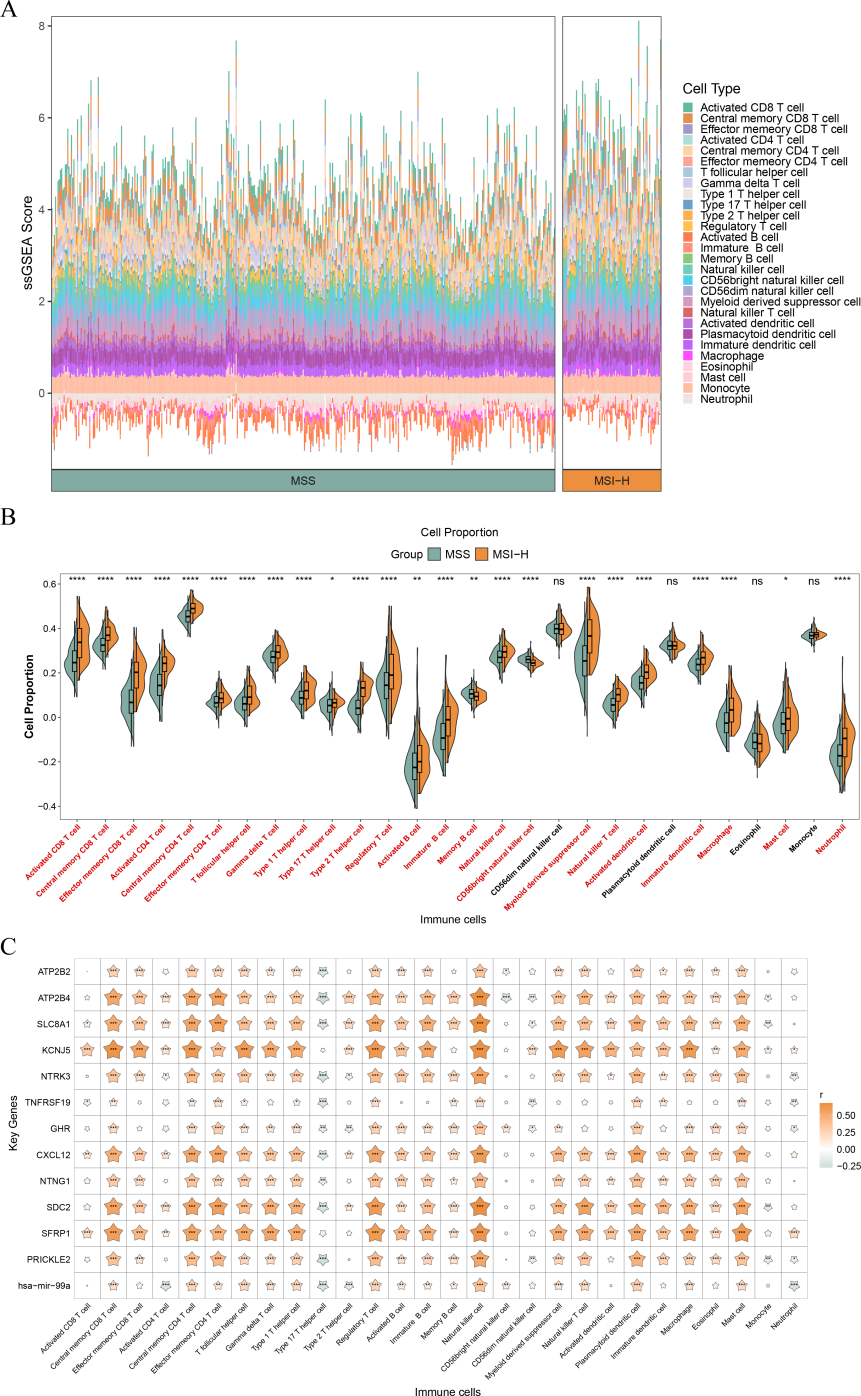
Differential immune landscape in CRC samples. **(A)** Immune infiltration landscape of CRC samples in MSS and MSI-H groups. **(B)** The difference of immune cells between MSS and MSI-H groups. **(C)** The correlation of immune cells and key genes. ns, no significance, *P < 0.05, **P < 0.01,***P < 0.001,****P < 0.0001.

Given the therapeutic relevance of immunotherapy in CRC, the TIME was characterized. **Figure 3A** mapped the 28 immune cell infiltration across all samples, and 24 types differed significantly between MSS and MSI-H groups (*P* < 0.05) (**Figure 3B**). Notable variations were observed in T helper 1 (Th1), Th17, Th2, effector memory CD8^+^ T, and gamma delta T cells. Correlation analysis further demonstrated that SDC2 (r = 0.679, *P* < 0.05) and hsa-miR-99a (r = 0.353, *P* < 0.05) showed the strongest positive correlation with Natural Killer (NK) cell infiltration. In contrast, ATP2B4 (r = -0.270, *P* < 0.05) and hsa-miR-99a (r = -0.212, *P* < 0.05) exhibited the highest negative correlation with Th17 cell infiltration (**Figure 3C**; **Supplementary Figures 2A–M**).

### 3.4 Single-cell analysis identifies T cells as a key population in CRC

**Figure 4:**
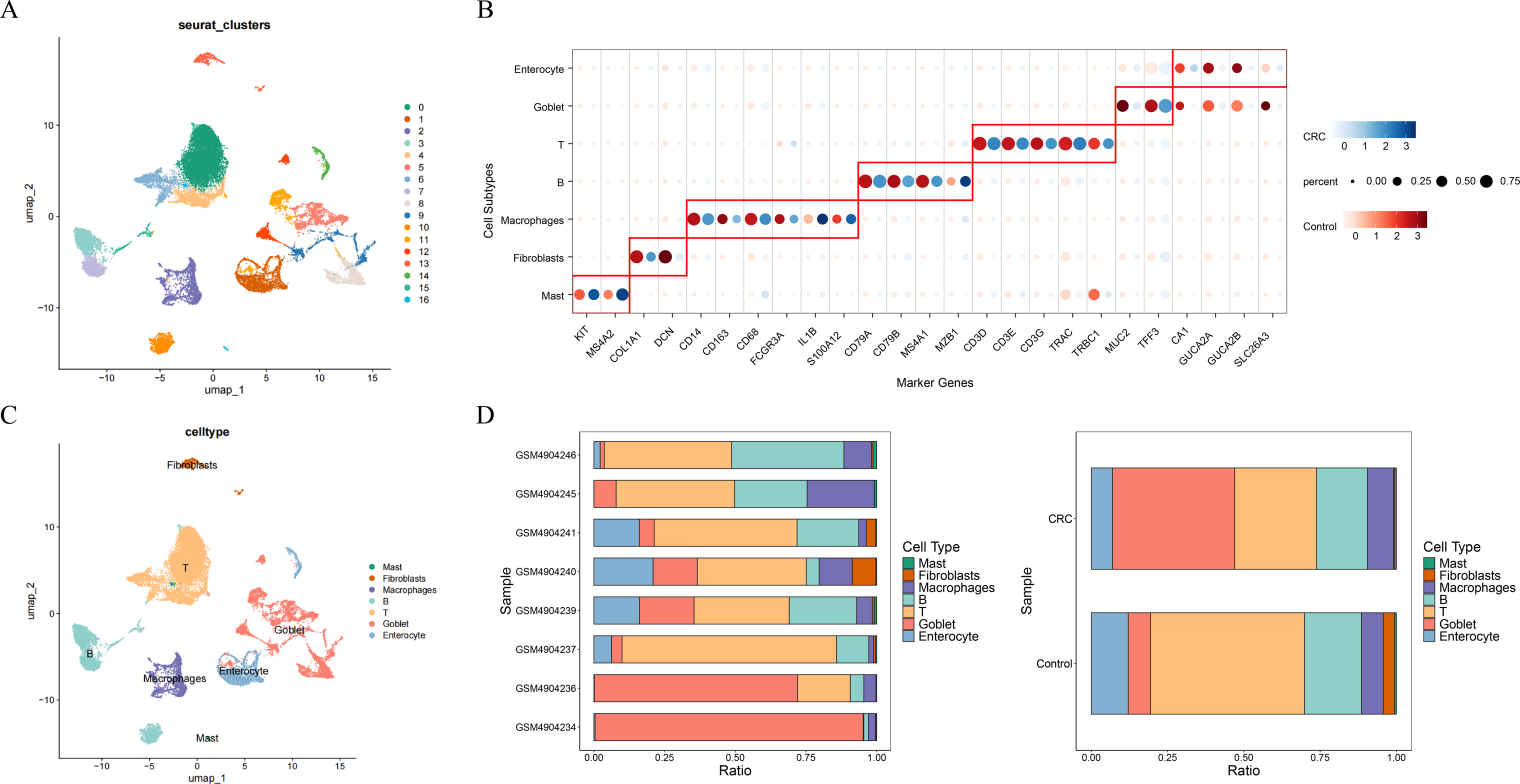
T cells were important in CRC samples. **(A)** The identification of cell clusters in single cell data. **(B)** Marker genes of cell subtypes. **(C)** The annotation of cell clusters. **(D)** The proportion of cell clusters in CRC and control samples.

To resolve cellular heterogeneity, the scRNA-seq dataset GSE161277 was analyzed. After quality control, 26,605 cells expressing 3,455 genes were retained and clustered into 17 distinct populations (**Figure 4A**; **Supplementary Figures 3A–C**). These clusters were annotated as seven major cell types based on canonical marker genes: enterocytes, goblet cells, T cells, B cells, macrophages, fibroblasts, and mast cells (**Figures 4B, C**). Proportion analysis revealed that T cells constituted the largest cellular compartment within the tumor microenvironment (**Figure 4D**). To further investigate T cell diversity, secondary sub-clustering was performed, resulting in 10 sub-clusters (**Supplementary Figure 4A**), which were classified into four subtypes: CD8+ T cells, central memory T (Tcm) cells, regulatory T (Treg) cells, and NKT cells (**Supplementary Figures 4B, C**). Among these, CD8+ T cells and Treg cells were significantly more abundant in CRC samples compared to controls (**Supplementary Figure 4D**).

**Figure 5:**
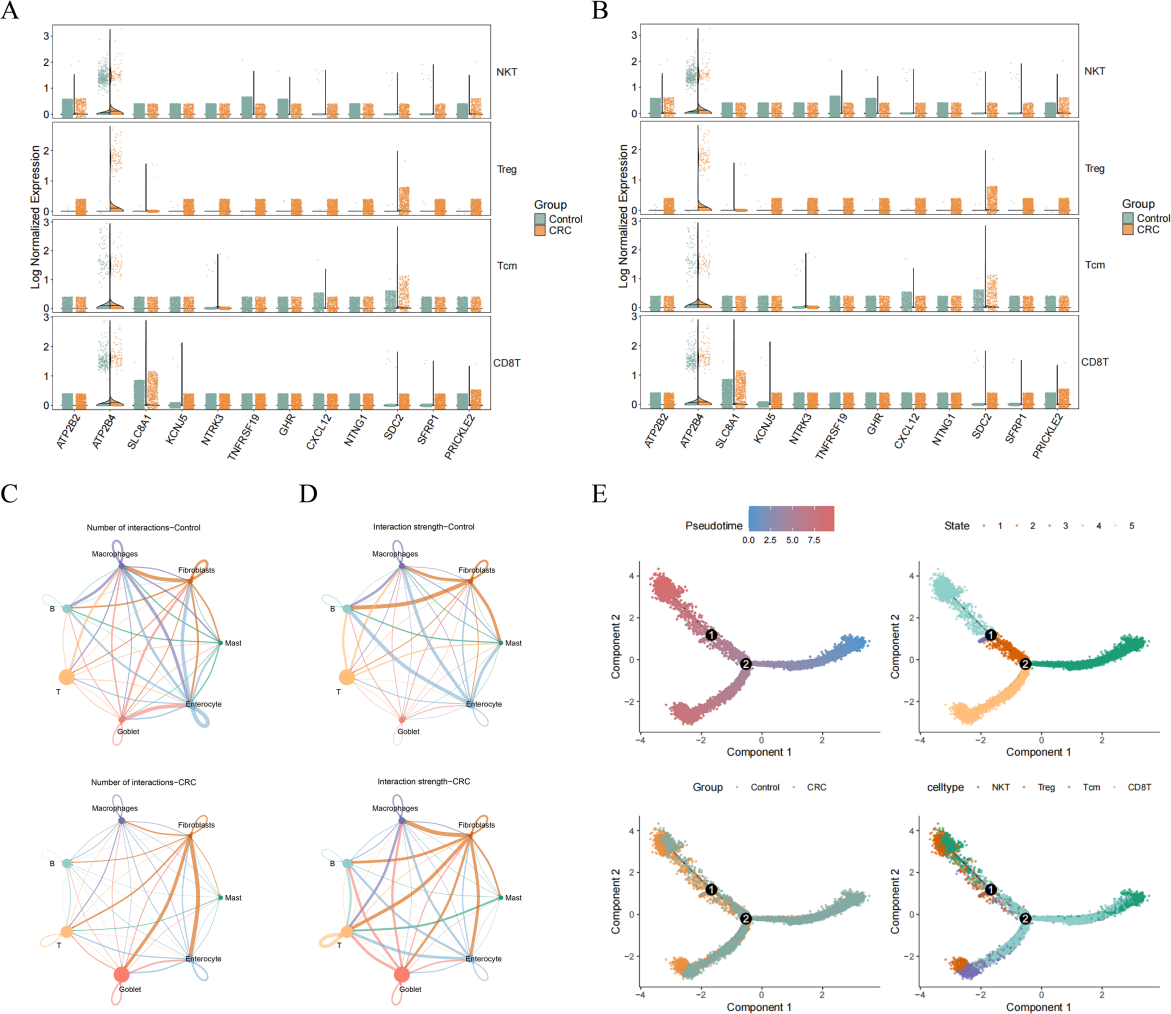
Cell communication and pseudo time-series analyses. **(A)** The expression of key genes in cell subtypes. **(B)** The expression of key genes in T cell subtypes. **(C, D)** Cell communication of cell subtypes in control **(C)** and CRC groups **(D)**. **(E)** The pseudo time-series analysis of T cells.

Evaluation of key gene expression across all cell types revealed that PRICKLE2 and SLC8A1 were differentially expressed in T cells between CRC and control samples (**Figure 5A**). Notably, PRICKLE2 expression was also significantly altered in NKT cells (**Figure 5B**). Cell-cell communication analysis indicated that all major cell subtypes exhibited more extensive interaction networks within the CRC microenvironment compared to normal tissue (**Figures 5C, D**; **Supplementary Figure 3D**). Pseudotime trajectory analysis of T cell differentiation suggested an advanced differentiation state in CRC, with NKT and Treg cells positioned at later stages, CD8+ T cells at early/middle stages, and Tcm cells spanning both early and late stages (**Figure 5E**). Among key genes, ATP2B4 was expressed throughout the T cell differentiation continuum (**Supplementary Figure 5**). Pathway activity scoring (AUCell) indicated that pathways such as “Endocrine and other factor-regulated calcium reabsorption” were most active in enterocytes (**Supplementary Figure 6A**). Within T cell subtypes, pathways like “Adrenergic signaling in cardiomyocytes” showed the highest activity in NKT cells (**Supplementary Figure 6B**).

### 3.5 hsa-miR-99a correlates with mTOR pathway activity in CRC

**Figure 6:**
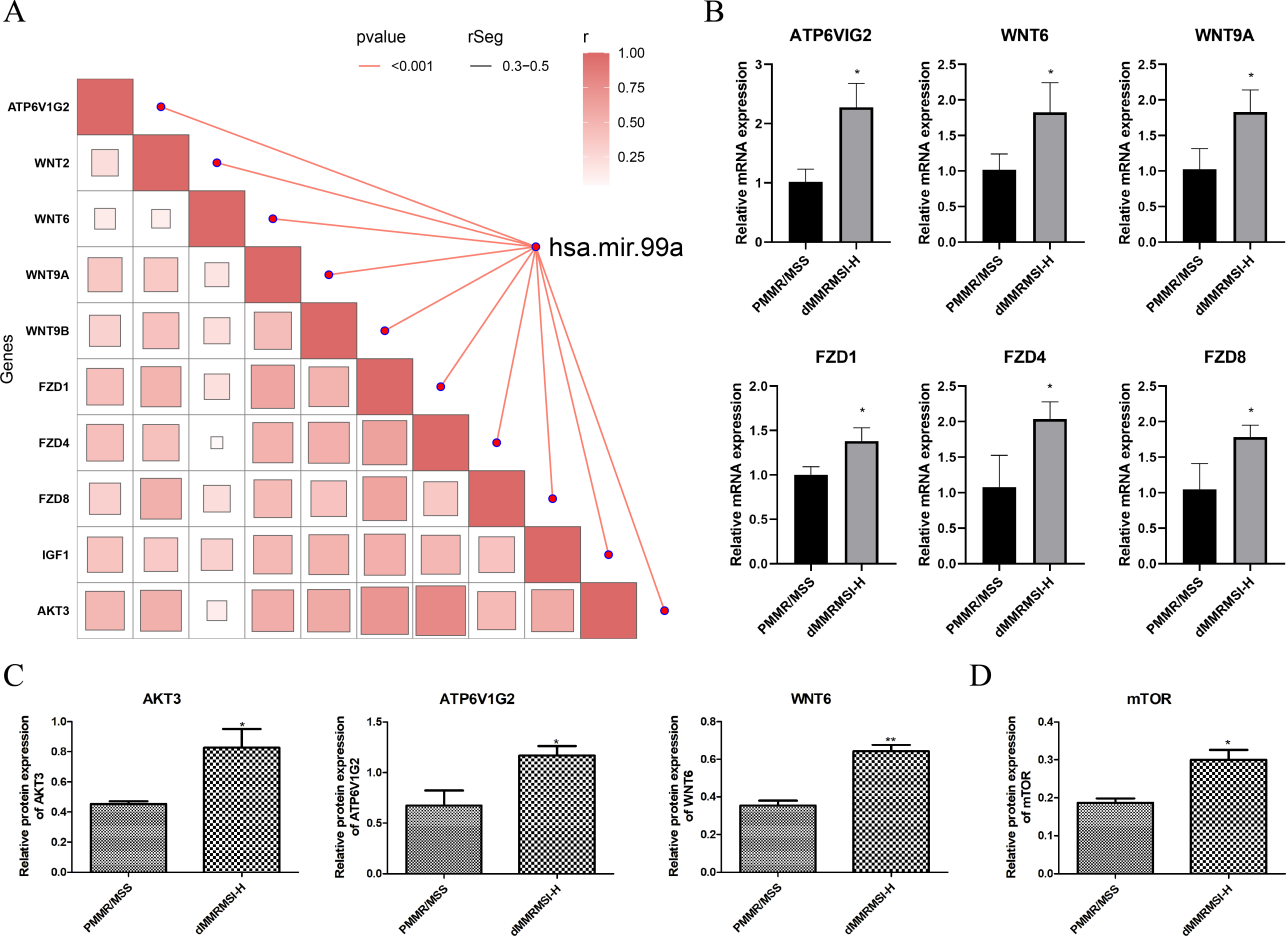
The functions of mTOR pathway in CRC samples. **(A)** The correlation of hsa-mir-99a with genes on mTOR pathways. **(B, C)** The expression of mTOR pathways-related genes at mRNA **(B)** and protein levels **(C)**. **(D)** The expression level of mTOR in the MSI-H group. Data are presented as mean ± SD from three independent biological replicates (n=3). *P < 0.05, **P < 0.01.

Given the established role of aberrant mTOR signaling in CRC progression, its interaction with hsa-miR-99a was investigated. Correlation analysis identified ten mTOR pathway-related genes whose expression strongly correlated with hsa-miR-99a levels (|r| > 0.3, p < 0.05): ATP6V1G2, WNT2, WNT6, WNT9A, WNT9B, FZD1, FZD4, FZD8, IGF1, and AKT3 (**Figure 6A**). RT-qPCR validation in patient blood samples confirmed significantly higher mRNA levels of ATP6V1G2, WNT6, WNT9A, FZD1, FZD4, and FZD8 in the MSI-H group compared to the MSS group (**Figure 6B**). Western blot analysis at the protein level confirmed significantly elevated expression of ATP6V1G2, WNT6, and AKT3 in MSI-H samples (**Figure 6C**). Consistent with elevated pathway activity, the protein level of the key kinase mTOR was also significantly higher in the MSI-H group (**Figure 6D**).

### 3.6 Immune phenotyping reveals an immunosuppressive shift in MSI-H CRC

**Figure 7:**
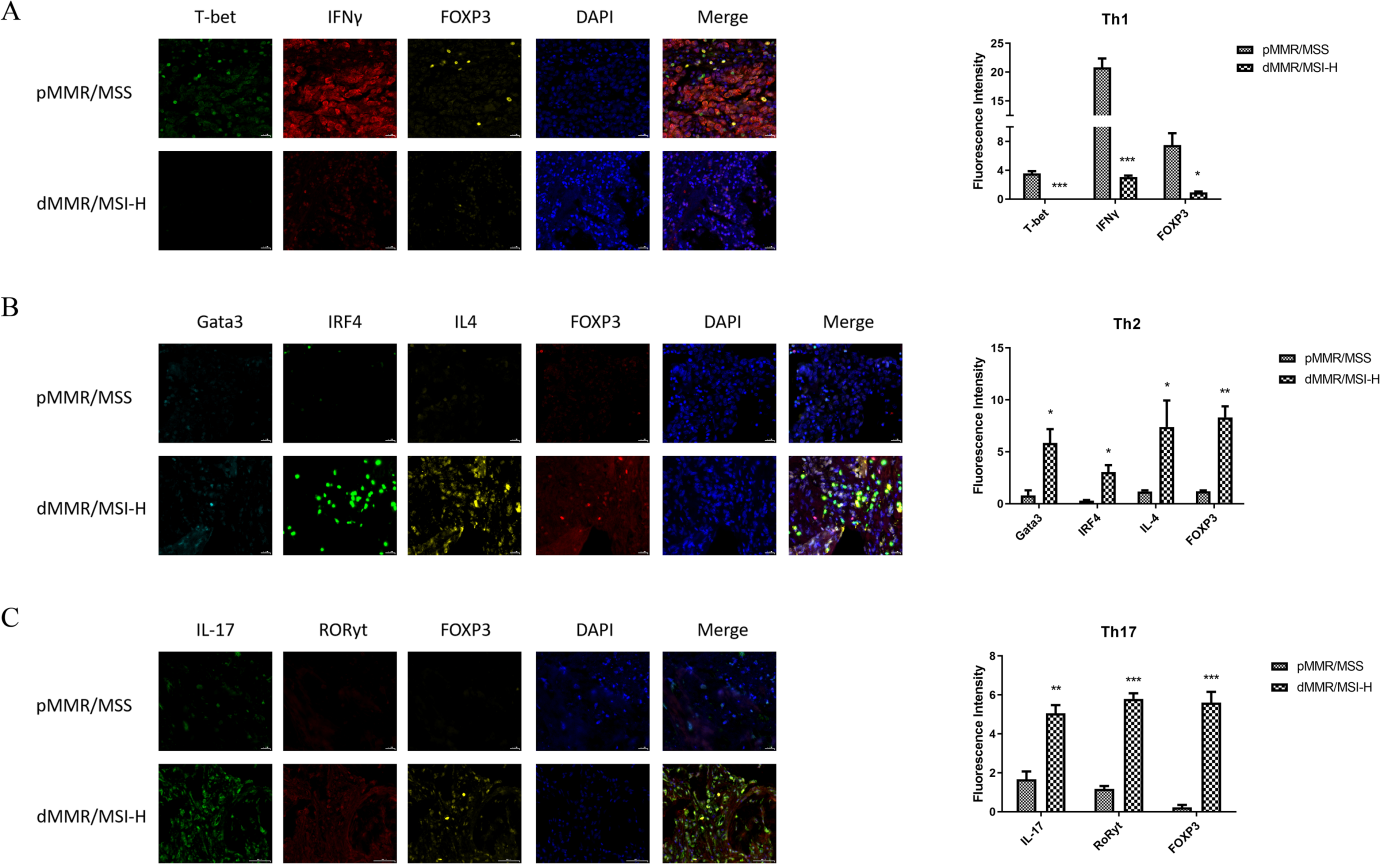
Immune penotyping analysis. **(A-C)** The expression of biomarkers for Th1 **(A)**, Th2 **(B)**, and Th17 cells **(C)**. *P < 0.05, **P < 0.01, ***P < 0.001.

Based on the differential infiltration of Th1, Th2, and Th17 cells, mIHC was performed to profile these subsets *in situ*. In MSI-H CRC tissues, the expression of Th1 cell biomarkers (T-bet, IFN-γ, and FOXP3) was significantly reduced compared to MSS tissues (**Figure 7A**). In contrast, biomarkers for Th2 cells (GATA3, IRF4, IL-4, and FOXP3) and Th17 cells (IL-17, RORγt, and FOXP3) were markedly elevated in the MSI-H group (**Figures 7B, C**). This pattern indicates a distinct immune context in MSI-H CRC, characterized by the suppression of pro-inflammatory Th1 responses and a concurrent increase in Th2 and Th17 cells, thereby fostering an immunosuppressive milieu that enables tumor immune escape.

## 4 Discussion

MiRNAs are recognized as pivotal post-transcriptional controllers that tune oncogenes and tumor suppressors, driving CRC pathogenesis (19). However, the specific biological functions of hsa-miR-99a in different MSI states (MSI-H and MSS) of CRC, its comprehensive regulatory network, and its clinical significance remain poorly understood. To systematically explore the molecular mechanisms of hsa-miR-99a in CRC, this study combined bioinformatics analysis with experimental validation, revealing that hsa-miR-99a is significantly upregulated in MSS compared to MSI-H tumors, and its elevated expression correlates with poorer patient prognosis. Additionally, a core set of 12 putative target genes (ATP2B2, ATP2B4, SLC8A1, KCNJ5, NTRK3, TNFRSF19, GHR, CXCL12, NTNG1, SDC2, SFRP1, and PRICKLE2) was identified, along with distinct immune microenvironment features associated with these genes. The expression of hsa-miR-99a was strongly correlated with the activity of the mTOR signaling pathway, with this association being particularly pronounced in the MSI-H subtype. These findings highlight the subtype-specific expression patterns, potential downstream targets, and signaling regulatory mechanisms of hsa-miR-99a in CRC.

Among the 12 key target genes of hsa-miR-99a, several have been implicated in the pathogenesis of CRC. ATP2B4, a plasma membrane calcium ATPase, has been linked to tumor location in CRC, suggesting its involvement in niche-specific tumor biology (20). SLC8A1 (also known as NCX1), a sodium-calcium exchanger, has recently emerged as a promising diagnostic biomarker; elevated plasma levels of SLC8A1 protein show potential for early-stage CRC detection (21, 22). The potassium channel gene KCNJ5 has been associated with CRC progression, with evidence suggesting that the transcription factor HSF4 may exert part of its oncogenic effect by modulating KCNJ5 expression (23). Similarly, NTRK3, a neurotrophic tyrosine receptor kinase, promotes CRC cell proliferation and survival, with its expression potentially regulated through non-canonical mechanisms, such as the intracellular domain of CD51 acting as a transcriptional co-activator (24). TNFRSF19 (TROY), this tumor necrosis factor receptor superfamily member alone predicts CRC outcome, underscoring its promise as a therapeutic target (25). CXCL12, a key chemokine regulating immune cell migration and tumor-stroma interactions, plays a critical role in CRC progression and metastasis (26, 27). SDC2, a syndecan involved in cell adhesion and signaling, is a well-established fecal DNA methylation biomarker for noninvasive CRC screening (28). SFRP1, a secreted antagonist of the Wnt pathway, is frequently silenced by promoter hypermethylation in colorectal adenomas and carcinomas, contributing to Wnt pathway activation (29). However, whether these genes are directly regulated by hsa-miR-99a in CRC—particularly across the distinct MSI subtypes of MSS and MSI-H—remains unclear. The core strength of this study lies in its novelty: for the first time, this study systematically revealed the direct regulatory relationship between hsa-miR-99a and these genes within the molecular context of CRC and clarified the differences in this regulation across distinct MSI subtypes. Furthermore, genes such as ATP2B2, GHR (Growth Hormone Receptor), NTNG1 (Netrin G1), and PRICKLE2 are less characterized in CRC. For example, PRICKLE2, a regulator of planar cell polarity, has mainly been studied in neurological contexts (30). Our single-cell analysis identified its differential expression in T and NKT cells, suggesting a previously unrecognized role in shaping the immune landscape of CRC. These genes, regulated through hsa-miR-99a, participate in the regulatory network governing CRC pathogenesis—an interaction not previously documented in the literature. This highlights the novelty of our findings and paves the way for further in-depth validation of the underlying functional mechanisms.

The interplay between tumor cells and the immune microenvironment is a critical factor in CRC progression and therapy response. Our immune infiltration analysis confirmed the well-established, more inflamed phenotype of MSI-H tumors compared to MSS, revealing significant differences across all 24 cell types examined. This study specifically focused on T helper subsets, where correlations with key genes were observed. The mIHC results demonstrated that MSI-H tumors exhibited a marked decrease in Th1 cell biomarkers (T-bet, IFN-γ), alongside an increase in Th2 (GATA3, IL-4) and Th17 (IL-17, RORγt) markers. This aligns with the concept of an “immunosuppressive switch.” Th1 cells, which are essential for anti-tumor cellular immunity, are suppressed, while Th2 cells (commonly associated with humoral and pro-tumorigenic responses) and Th17 cells (which can have both pro- and anti-tumor functions but often promote inflammation and angiogenesis in CRC) are enriched (31, 32). The negative correlation between hsa-miR-99a/ATP2B4 and Th17 infiltration further supports the idea that the hsa-miR-99a regulon may indirectly influence this immune axis. In summary, despite the high immune infiltration in MSI-H tumors, the tumor microenvironment shows a shift toward a Th2/Th17-dominant suppressive phenotype. This may represent one mechanism underlying adaptive immune resistance in these tumors and offers new theoretical support for future combination immunotherapies targeting the Th2/Th17 pathway.

The mTOR pathway is a central regulator of growth signals, and its hyperactivation is a common feature in CRC. This study reveals a significant positive correlation between hsa-miR-99a expression and mTOR signaling in CRC (particularly in the MSI-H subtype), uncovering a novel regulatory relationship. Specifically, the expression of several mTOR pathway-related genes (ATP6V1G2, WNT2/6/9A/9B, FZD1/4/8, IGF1, AKT3) exhibited high synchrony with hsa-miR-99a. Experimental validation in patient samples further revealed that several of these genes, including ATP6V1G2, WNT6, and AKT3, were significantly upregulated at the protein level in MSI-H tumors. Concurrent high mTOR protein expression was observed in these samples, confirming heightened mTOR pathway activity in this subtype. Mechanistically, these genes form an interactive network that exerts a synergistic effect on mTOR signaling: ATP6V1G2, a component of the V-ATPase complex, acidifies intracellular compartments, playing a critical role in nutrient sensing and signaling to mTORC1 (33); WNT6 and its receptors FZD1/4/8 activate the canonical Wnt/β-catenin pathway, which extensively interacts with mTOR signaling to drive proliferation and stemness (34); IGF1, a potent upstream activator of the PI3K/AKT/mTOR axis, further amplifies this network; and the significant upregulation of AKT3, a key mTOR activator, further strengthens the connection (35). While hsa-miR-99a is widely reported to repress mTOR signaling in other cellular contexts, our correlation data point to a potential positive regulatory loop or a coordinated upregulation pattern of hsa-miR-99a and mTOR signaling in specific CRC subtypes, most notably in the MSI-H subtype. This indicates a subtype-specific mechanism where hsa-miR-99a, through indirect networks or by targeting upstream inhibitors, may contribute to mTOR pathway dysregulation, driving the distinct biology of MSI-H cancers.

However, this study has some significant limitations. Firstly, although our analysis mainly focused on the mTOR signaling pathway, the other significantly enriched pathways identified in the study (such as the calcium signaling pathway) still need to be verified through experiments, and their potential interactions with the mTOR axis and the potential regulatory mechanisms in CRC have not been fully elucidated. More importantly, the direct regulatory relationships proposed in the identified axes lack strict functional validation experiments, including functional enhancement/reduction experiments and luciferase reporter experiments, which are crucial for confirming the direct molecular interactions between key components. Additionally, our clinical validation was based on a single-center cohort, which may limit the general applicability of our findings and restrict their external validity for a broader CRC patient population. Therefore, these unresolved aspects will become the core focus of our subsequent in-depth research. We plan to conduct systematic *in vitro* and *in vivo* functional validation experiments to strictly verify the direct regulatory relationship between hsa-miR-99a and its key target genes. Moreover, we will establish partnerships with multiple medical centers to expand the clinical validation sample group, enabling us to verify our current research results in a large-scale, multi-center cohort of colorectal cancer patients. This expanded validation will not only confirm the reproducibility of our results but also significantly enhance the external validity and clinical translational potential of our research conclusions.

## 5 Conclusion

In conclusion, this study provides a comprehensive analysis of the multifaceted role of hsa-miR-99a in CRC, bridging genomic regulation, immune microenvironment remodeling, and oncogenic pathway activity. A novel regulatory network centered on hsa-miR-99a is proposed, involving key genes like SLC8A1 and TNFRSF19, which have direct clinical relevance and influence immune cell dynamics. Notably, a strong association is established between hsa-miR-99a expression and mTOR pathway activity in CRC, with ATP6V1G2 and WNT6 identified as potential key effector molecules in MSI-H tumors. These findings position hsa-miR-99a and its downstream targets, particularly within the mTOR axis, as promising candidates for the development of subtype-specific diagnostic biomarkers or therapeutic strategies.

## Data Availability

The original contributions presented in the study are included in the article/**Supplementary Material**. Further inquiries can be directed to the corresponding author.
