# Supplementary material for: Hsa-miR-99a deficiency contributes to MSI-H colorectal cancer progression by activating the mTOR pathway and inducing Th1/Th2 imbalance
Source: Front Immunol. 2026 Mar 17;17:1796084. doi: 10.3389/fimmu.2026.1796084 (PMC13036217; doi:10.3389/fimmu.2026.1796084)
Supplement: Supplementary file 1 [file Table1.docx]

**
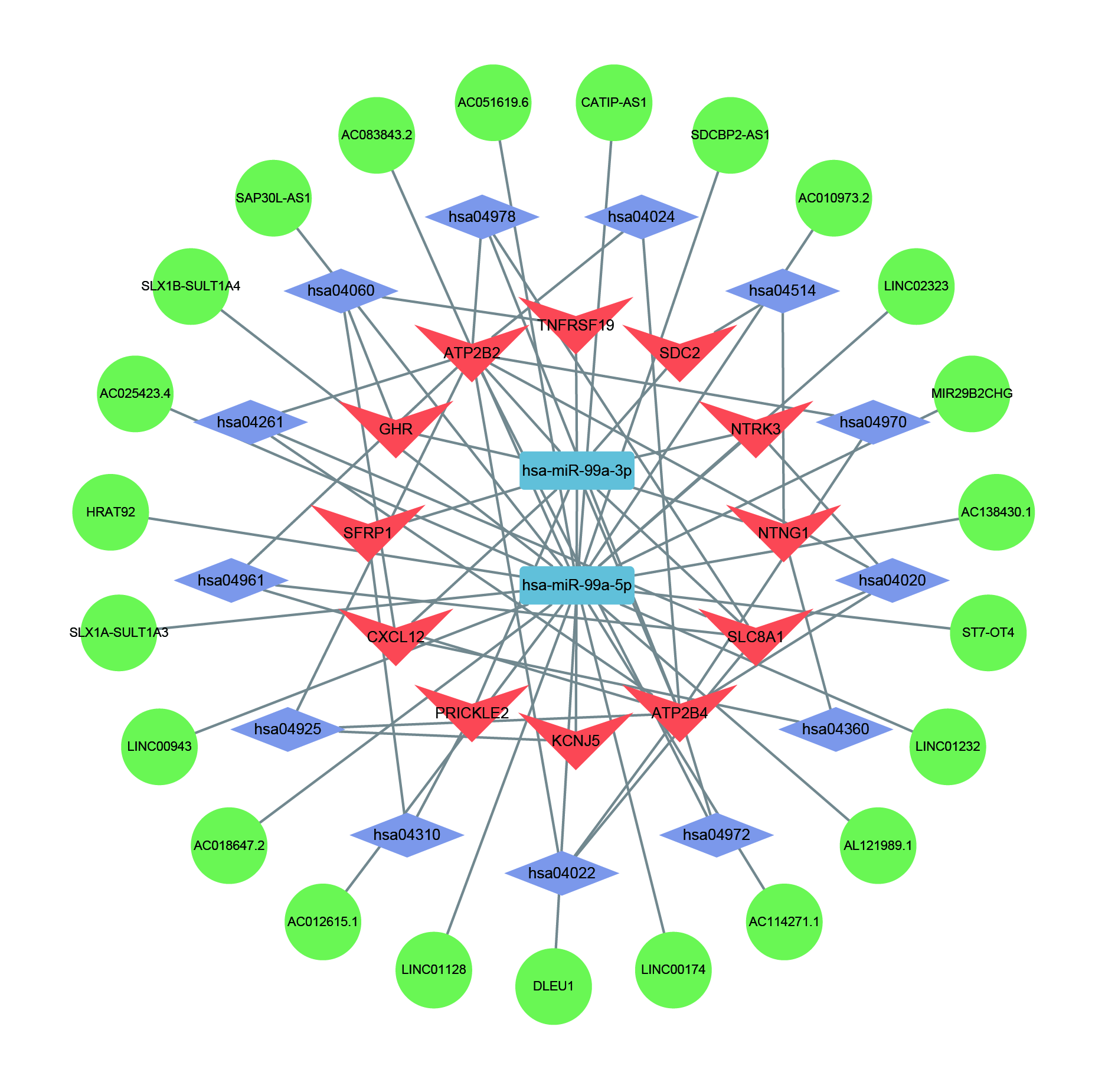
**

**Supplementary Figure 1** **The regulatory network of mRNA, microRNA (miRNA), long noncoding RNA (lncRNA).**

**
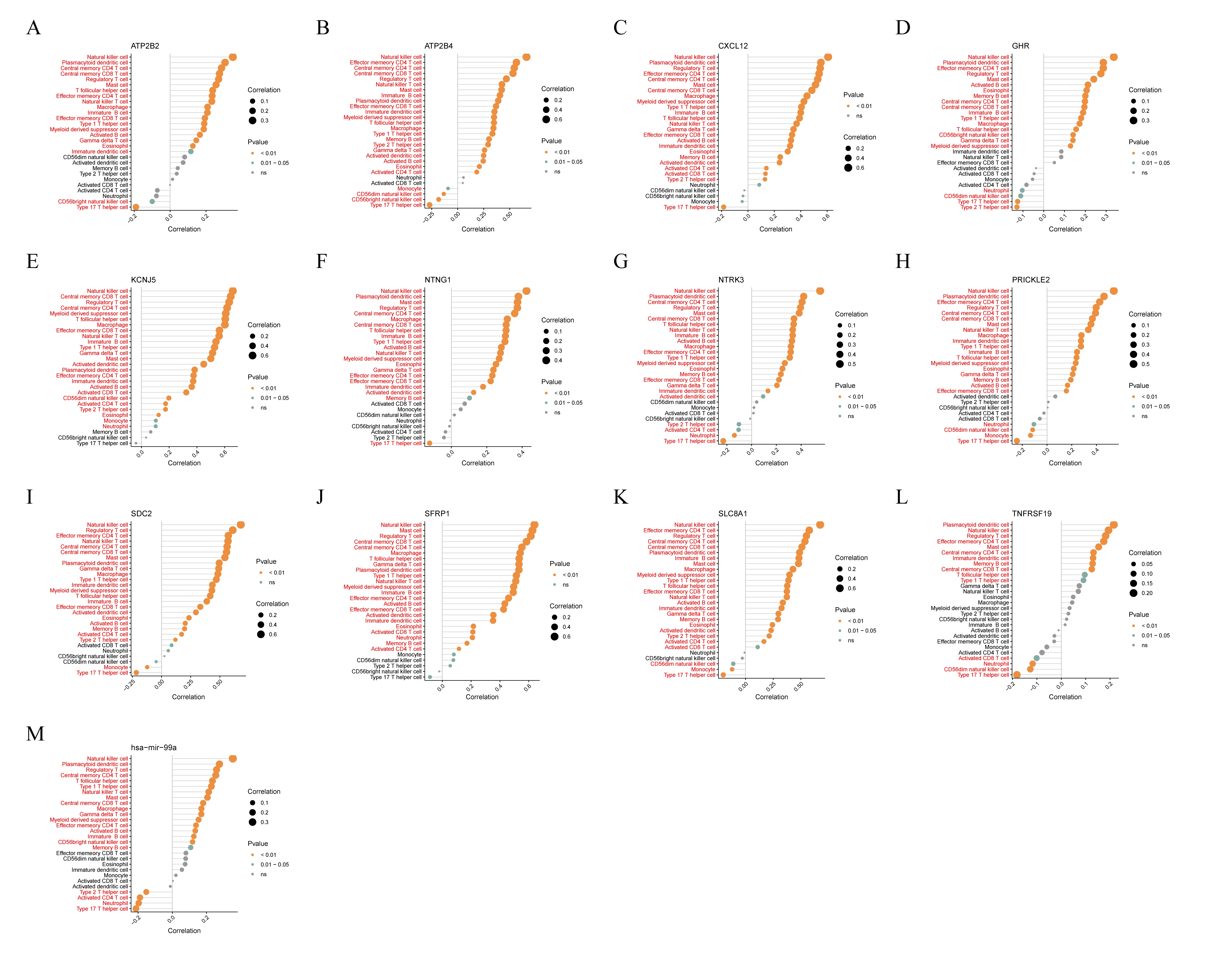
**

**Supplementary Figure 2 The correlation of immune cells with key genes and hsa-mir-99a. (A-M)** The correlation of immune cells with ATP2B2 **(A)**, ATP2B4 **(B)**, CXCL12 **(C)**, GHR **(D)**, KCNJ5 **(E)**, NTNG1 **(F)**, NTRK3 **(G)**, PRICKLE2 **(H)**, SDC2 **(I)**, SFRP1 **(J)**, SLC8A1 **(K)**, TNFRSF19 **(L)**, and hsa-mir-99a **(M)**.

**
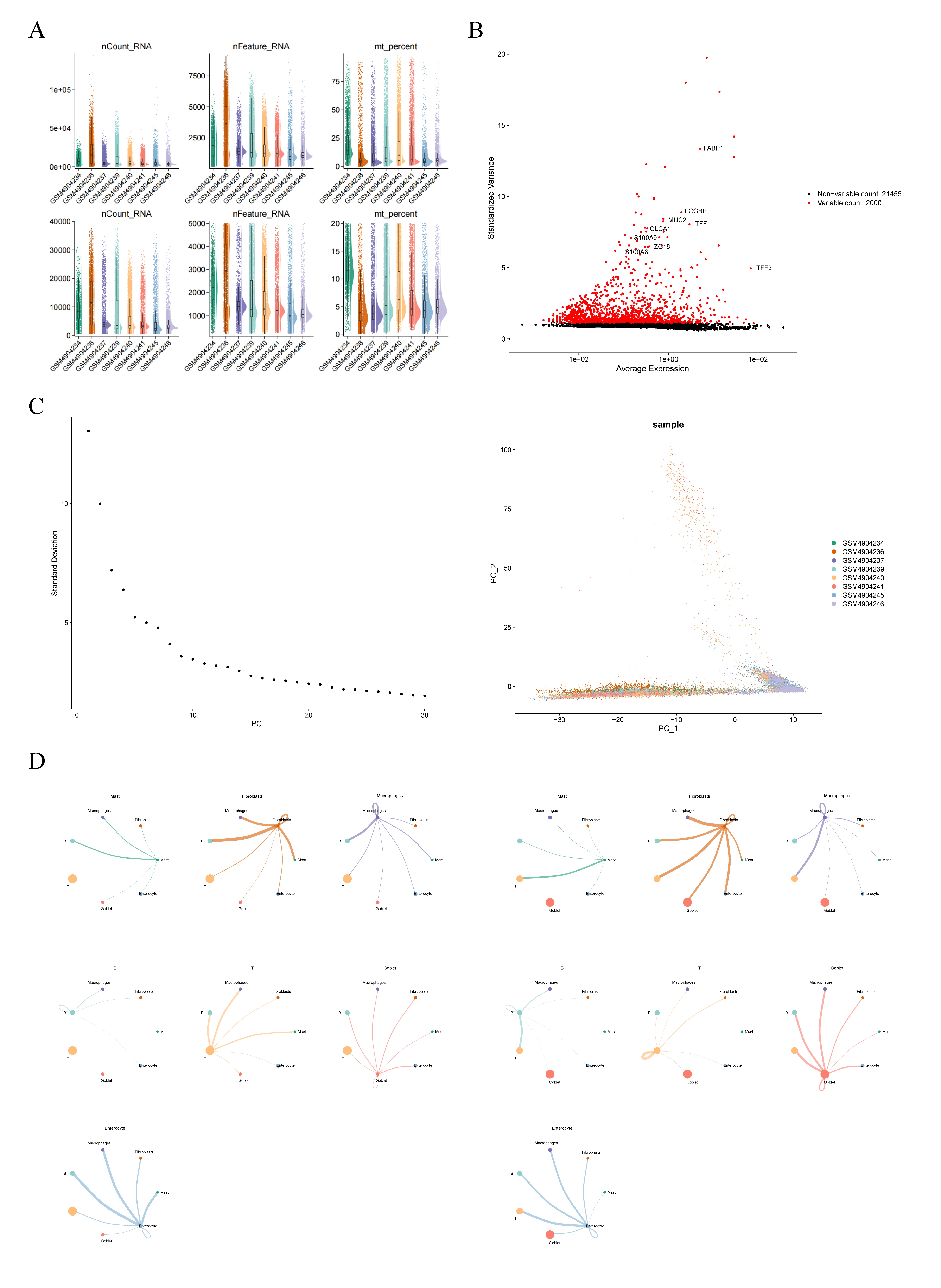
**

**Supplementary Figure 3 single-cell RNA sequencing (scRNA-seq) analysis in colorectal cancer (CRC) samples. (A)** The quality control in single cell data. **(B)** Top 2,000 high variable genes of CRC samples. **(C)** Top 30 principle components in CRC samples. **(D)** The cell communication of each cell subtype with other cells.


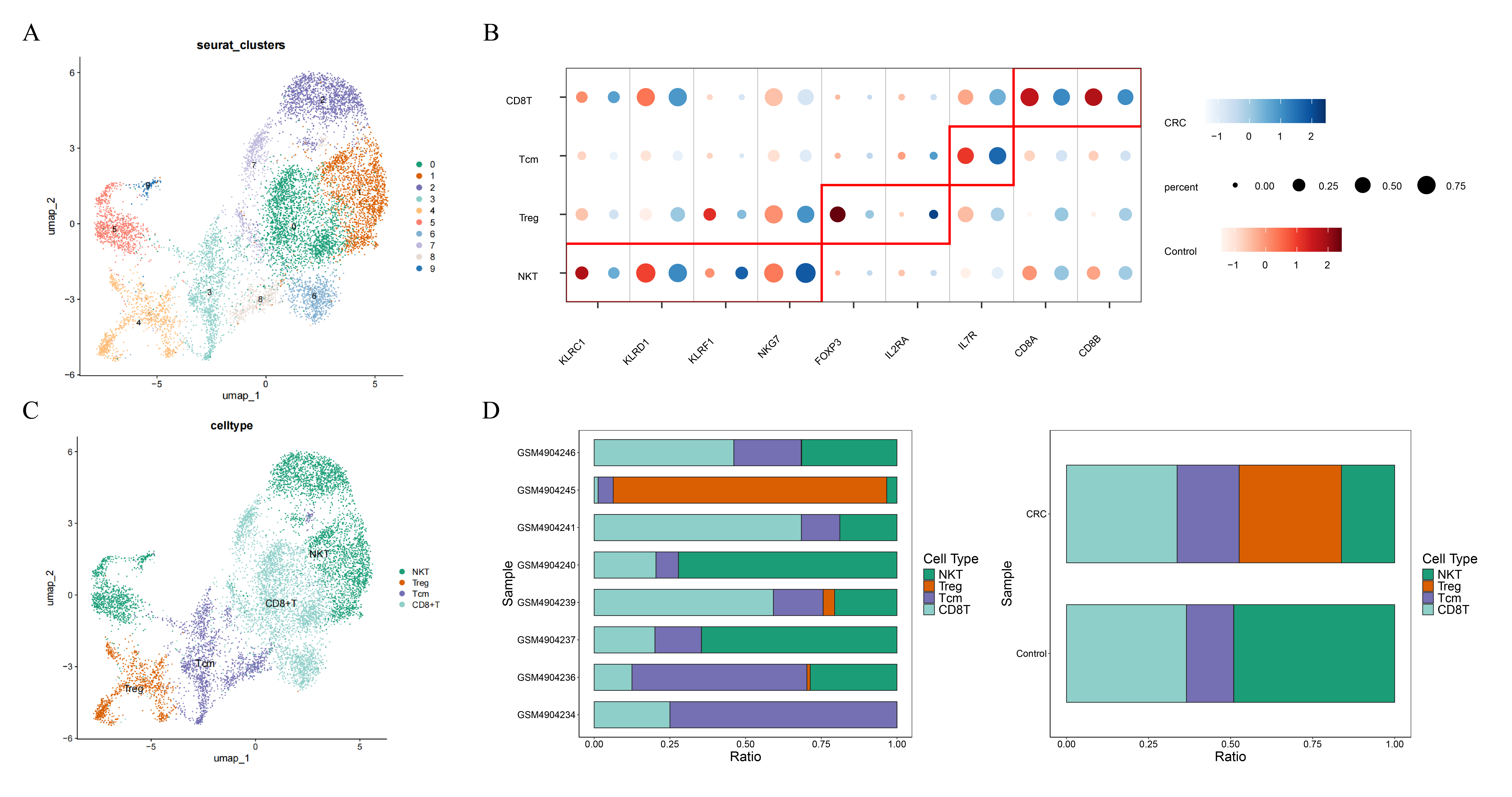


**Supplementary Figure 4 T cells were important in CRC samples. (A)** The identification of T cell clusters. (**B**) Marker genes of T cell subtypes. (**C**) The annotation of T cell subtypes. (**D**) The proportion of T cell subtypes in CRC and control samples.


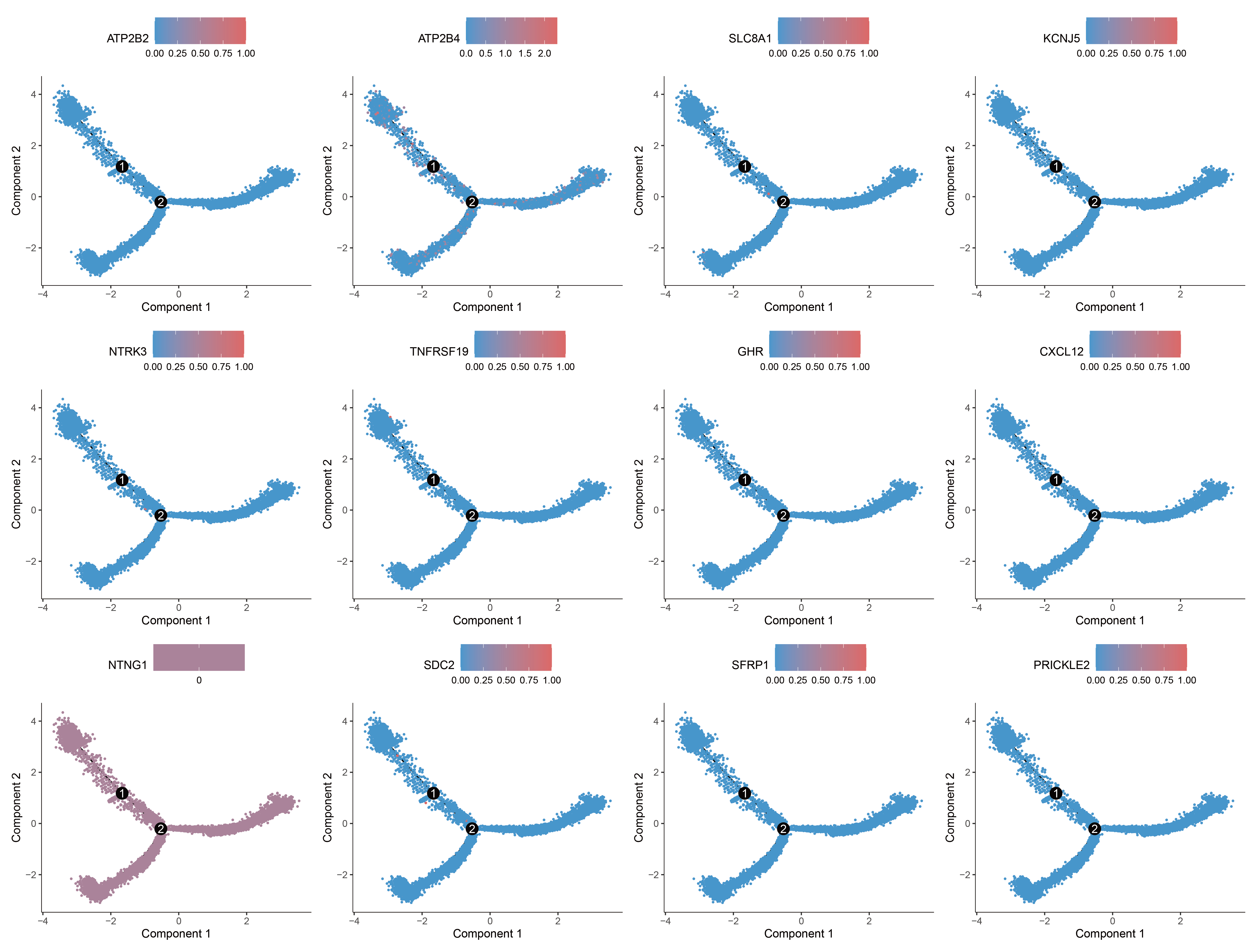


**Supplementary Figure 5 The expression of key genes in T cell differentiation.**

**
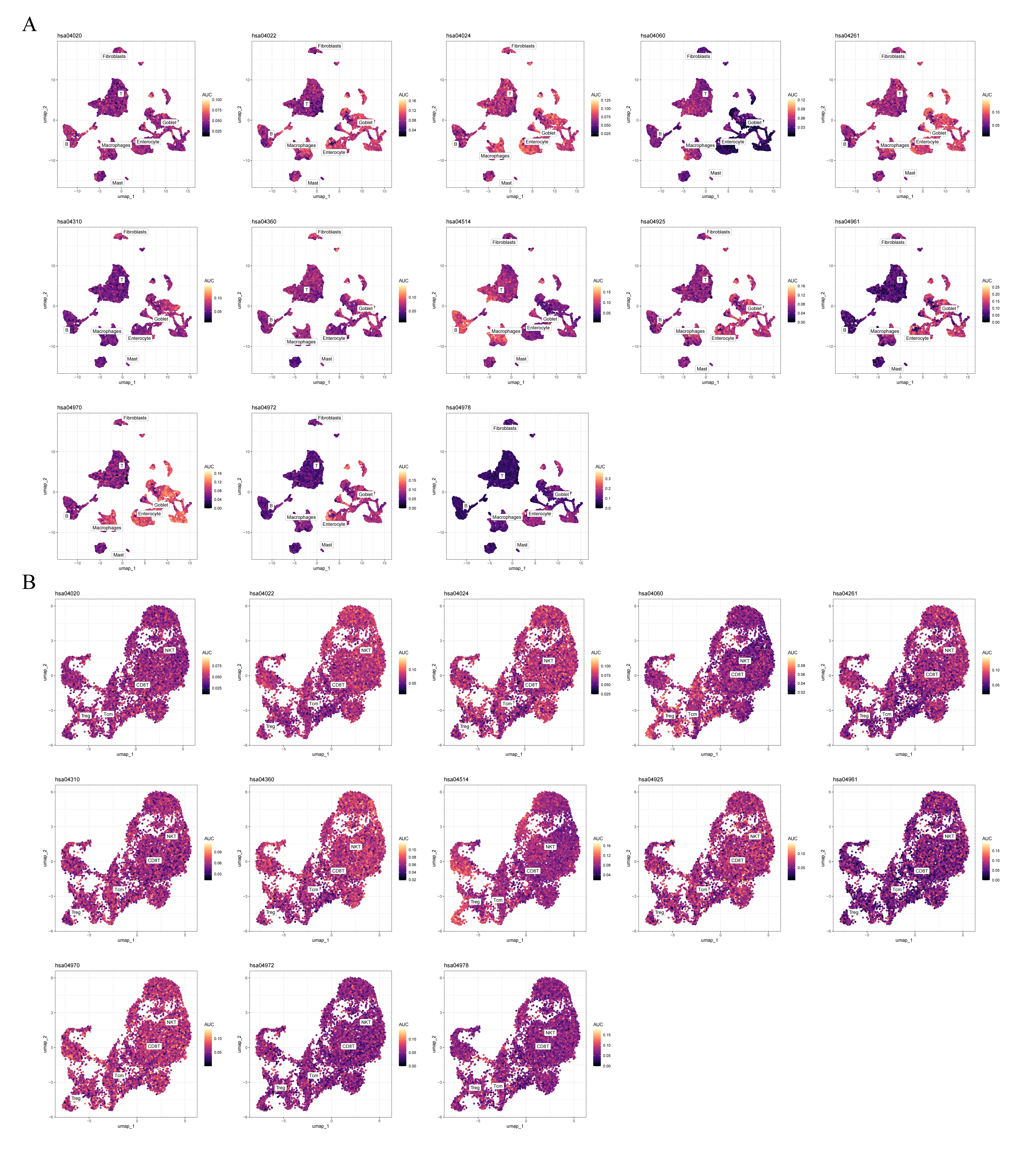
**

**Supplementary Figure 6 AUCell analysis of Kyoto Encyclopedia of Genes and Genomes (KEGG) pathways. (A-B)** The AUC score of KEGG pathways in cell subtypes **(A)** and T cell subtypes **(B)**.

**Supplementary Table 1. The sequences for primers**

| **Primers** | **Sequence (5’-3’)** |
| --- | --- |
| GAPDH | F:5**’**-CTGGGCTACACTGAGCACC-3’ |
|  | R:5**’**-AAGTGGTCGTTGAGGGCAATG-3’ |
| AKT3 | F:5**’**-AATGGACAGAAGCTATCCAGGC-3’ |
|  | R:5**’**-TGATGGGTTGTAGAGGCATCC-3’ |
| ATP6VIG2 | F:5**’**-CGGCGACTGAAGCAGGCTA-3’ |
|  | R:5**’**-AGACCATGCCAAGAAGCTGG-3’ |
| WNT2 | F:5**’**-GATGCGTGCCATTAGCCAG-3’ |
|  | R:5**’**-AGATTCCCGACTACTTCGGAG-3’ |
| WNT6 | F:5**’**-GGCAGCCCCTTGGTTATGG-3’ |
|  | R:5**’**-CTCAGCCTGGCACAACTCG-3’ |
| WNT9A | F:5**’**-GGCAAGCATCTGAAGCACAAG-3’ |
|  | R:5**’**-GCAGAAGCTAGGCGAGTCA-3’ |
| WNT9B | F:5**’**-TGTGCGGTGACAACCTCAAG-3’ |
|  | R:5**’**-ACAGGAGCCTGATACGCCAT-3’ |
| FZD1 | F:5**’**-ATCGAAGCCAACTCACAGTATTT-3’ |
|  | R:5**’**-CACGTTGTTAAGCCCCACG-3’ |
| FZD4 | F:5**’**-GTCTTTCAGTCAAGAGACGCTG-3’ |
|  | R:5**’**-GTTGTGGTCGTTCTGTGGTG-3’ |
| FZD8 | F:5**’**-TACAACCGCGTCAAGACAGG-3’ |
|  | R:5**’**-CCATGTCGATAAGGAAGGTGGAG-3’ |
| IGF1 | F:5**’**-TTGCTCTCAACATCTCCCATCT-3’ |
|  | R:5**’**-ATGGTGTGCATCTTCACCTTCA-3’ |
| miR-99a | F:5**’**-TTGGGTGAAATATATTGTGCGTCTC-3’ |
|  | R:5**’**-GAGTCCTGGACACCCAACTACAAG-3’ |
| U6 | F:5**’**-CTCGCTTCGGCAGCACA-3’ |

**Supplementary Table 2. Information related to genes and pathways**

| **Pathways** | **Genes** |
| --- | --- |
| Endocrine and other factor-regulated calcium reabsorption | ATP2B2/ATP2B4/SLC8A1 |
| Mineral absorption | ATP2B2/ATP2B4/SLC8A1 |
| Aldosterone synthesis and secretion | ATP2B2/ATP2B4/KCNJ5 |
| Calcium signaling pathway | ATP2B2/ATP2B4/SLC8A1/NTRK3 |
| Adrenergic signaling in cardiomyocytes | ATP2B2/ATP2B4/SLC8A1 |
| cGMP-PKG signaling pathway | ATP2B2/ATP2B4/SLC8A1 |
| Salivary secretion | ATP2B2/ATP2B4 |
| Cytokine-cytokine receptor interaction | TNFRSF19/GHR/CXCL12 |
| Pancreatic secretion | ATP2B2/ATP2B4 |
| Cell adhesion molecules | NTNG1/SDC2 |
| Wnt signaling pathway | SFRP1/PRICKLE2 |
| Axon guidance | NTNG1/CXCL12 |
| cAMP signaling pathway | ATP2B2/ATP2B4 |
| Endocrine and other factor-regulated calcium reabsorption | ATP2B2/ATP2B4/SLC8A1 |
